# Group-Learning Activities and Nurses Internalization of Evidence-Based Practices: Secondary Analysis of a Cross-Sectional Study in Hospital Wards

**DOI:** 10.1155/jonm/6080964

**Published:** 2025-03-03

**Authors:** Keiko Ishii, Yukie Takemura, Aya Kitamura

**Affiliations:** ^1^Department of Home Health and Palliative Care Nursing, Graduate School of Health Care Sciences, Institute of Science Tokyo, Tokyo, Japan; ^2^Nursing Department, The University of Tokyo Hospital, Tokyo, Japan; ^3^Department of Gerontological Nursing, Ishikawa Prefectural Nursing University, Ishikawa, Japan

## Abstract

**Aim:** This study is a secondary analysis aimed at verifying the relationship between organizational learning activities for evidence-based practices (EBPs) in hospital wards and nurses' internalization of those EBPs and analyzing the contextual effects.

**Methods:** This study used data from a previous study which was conducted to develop the group organizational learning activity inventory and used the same sampling method. The participants were asked about the extent of their internalization of EBPs, the organizational learning activities in their ward, and individual and ward characteristics. This study employed two-level hierarchical linear modeling with nurses' internalization of EBPs as the objective variable, eight factors of the Group Organizational Learning Activity (GOLA) Inventory as the explanatory variable, and individual and ward characteristics as control variables. Nurses' individual scores for the eight factors of the GOLA Inventory were analyzed by centering within clusters, and the wards' mean GOLA Inventory scores were also examined. To show the effectiveness of concrete activities for the internalization of EBPs, we calculated the contextual effects of the wards' organizational learning activities on the internalization of EBPs.

**Results:** As in the primary analysis, a total of 422 nurses from 56 wards in 12 hospitals responded to the survey and 360 nurses from 48 departments were included in this secondary analysis. Although the mean scores of all eight factors of the GOLA Inventory (ward level) were significantly positively associated with the internalization of EBPs, the contextual effect of each factor differed.

**Conclusion:** Rather than creating an EBP team, specific ward-level activities, such as those designed to ensure that the staff can understand why EBPs are implemented and encourage them to take ownership of EBPs, are necessary for nurses' internalization of EBPs. Based on the results of this research, hospital or ward managers, as well as staff involved in introducing new practices, can implement efforts to promote nurses' internalization of EBPs.

## 1. Introduction

To implement evidence-based practices (EBPs), nurses should understand the rationale and value of EBPs and change their way of thinking. Furthermore, the continuous implementation of EBPs involves a change not only in the behavior of clinicians but also in their mindsets and attitudes toward EBPs [1]. Previous research has shown that knowledge and attitudes toward EBPs are important factors in promoting the implementation of EBPs [2–4]. Nurses generally show a positive attitude toward EBPs [5, 6]. Some nurses, however, view EBPs as tasks ordered by hospitals [7]. This perception, which reflects a lack of understanding of the meaning and value of EBPs, might impede their sustained implementation. Understanding the rationale and value of EBPs is essential for their continuous implementation. In clinical settings, finding new ways of fostering nurses' understanding of the rationale and value of an EBP is thus an urgent issue.

Internalization is the process through which individuals transform explicit knowledge into practical knowledge [8], recognize its value [9], and subsequently use it in their daily activities [10]. Research outside the hospital setting shows that employees who internalize organizational practices are more likely to engage in those practices [10]. Internalization could thus serve as a key influencing factor behind nurses' future adherence to an EBP [11] and promote their continuous implementation of it.

Previous research has identified the organizational- and individual-level antecedents to the internalization of knowledge, including supportive and respectful organizational environments [12], senders' capacity to disseminate knowledge, receivers' capacity to absorb it [9], and learning motivation [13]. Furthermore, individuals' internalization of knowledge is influenced by the group(s) to which they belong [14]. The characteristics of the group in which the EBP is implemented, such as organizational climate and workplace environment, also relate to the implementation of EBPs [15–17]. Since EBPs are often implemented at the department or ward level in hospitals, the specific characteristics of departments and wards could influence nurses' internalization. Therefore, group-level organizational learning could be an effective way for nurses to internalize EBPs.

Group learning is part of the organizational learning process, which involves three levels that proceed in the order of individuals, groups, and organizations. Generally, the knowledge acquired by individuals is transferred to the group and then shared and translated into organizational routines [18]. Conversely, organizational learning also occurs when the knowledge institutionalized by an organization is transferred to the group; individuals then use the knowledge through interpretation and integration within the group and change their perceptions of and behaviors toward this knowledge [18]. This learning process follows the sequence of learning from the organization to the group and then to individuals (known as the feedback process) [18].

By engaging in group-level organizational learning through this feedback process, nurses could be involved in EBPs in a hospital's departments or wards, thereby increasingly internalizing them. Previous research shows that team learning promotes the establishment of common knowledge, shared values, and shared understanding about knowledge among team members [19, 20] and improves the critical thinking of nurses [21]. In a study conducted prior to the present study, which examined groups' organizational learning as a variable, groups' organizational learning in a ward was not associated with the nurses' internalization of EBPs [22]. Conversely, nurses' internalization of EBPs was significantly associated with the way they sustain those EBPs. By clarifying the association between more concrete group-level organizational learning and nurses' internalization of EBP, we could suggest specific ward-level activities to encourage individual nurses' internalization of EBP. If group-level organizational learning can foster the internalization of EBPs, by doing it at the entire departments or wards, departments and wards can promote nurses' understanding of the value of EBPs and cultivate positive attitudes toward them. This would prevent nurses from implementing EBPs superficially and facilitate safer and higher quality practices. Based on the foregoing information, to uncover concrete group activities that are effective for nurses' internalization, this study aimed to verify the relationship between various organizational learning activities in departments and wards and nurses' internalization of the EBPs institutionalized by hospitals.

## 2. Materials and Methods

The data for this study were collected concurrently with the development of the Group Organizational Learning Activity (GOLA) Inventory [23]. Therefore, the data of the hospitals and selected EBPs used in this study are the same as those reported in the study on the development of the inventory [23]. Furthermore, this study was conducted to complement the results of the hypothesis testing in the previous study [22]. However, the objectives and methods of this study are significantly different from the previous one. For the previous research, data were collected to elucidate the relationships among GOLA, nurses' and groups' internalization of EBPs, and the nurses' sustainment of EBPs. Since the core measurements of the primary study are the same as those of this study, conceptual slippage in proxy measures could be prevented by conducting the secondary analysis using primary data [24]. With such a secondary analysis, hypotheses that could not be verified through primary research can be verified in more detail [25].

### 2.1. Data Collection and Selection of EBPs

As this examined the concrete effects of group-level organizational learning activities, we calculated that the minimum sample size required for multilevel analyses focusing on fixed effects in cross-sectional studies would be 30 groups (or departments/wards in this case) [26] with 30 individuals in each group [27]. Participant selection is referenced in Figure 3 in [22]. EBPs that asked participants about the degree of internalization are detailed in the study of Ishii et al. [23].

### 2.2. Measures

#### 2.2.1. Internalization of EBPs

EBP internalization scale [22] was used to measure nurses' internalization of EBP. This scale defines the internalization of EBPs as the medical staff understanding of the rationale and value of those EBPs, accepting their importance, and incorporating them into their practice with a positive attitude and sense of responsibility. The scale consists of 10 items under two factors—understanding the significance of EBP (four items) and understanding the reason and purpose of EBP (six items). Each item was rated on a five-point Likert scale, ranging from 1 (*not at all*) to 5 (*very well*). Cronbach's alpha values for the factors were 0.92 and 0.93, respectively. This used the sum of the means of the two factors divided by the number of factors. A higher score indicated that the individual had internalized the target EBP. (You can refer to the items in Appendix A [22]).

#### 2.2.2. GOLAInventory

The GOLA Inventory [23] was used to measure the organizational learning activities in the departments/wards of the sampled hospitals. This scale consists of eight factors with 40 items in total: organizing a team to lead the EBP in the unit (Factor 1); evaluating the implemented EBP from multiple angles (Factor 2); ensuring that the staff can acquire common knowledge of the EBP (Factor 3); ensuring that the staff can understand why the EBP is being implemented (Factor 4), ensuring that the staff can implement the EBP in a unified manner (Factor 5); sharing the significance of EBP implementation with the unit and staff (Factor 6); encouraging the staff to better implement the EBP within the unit (Factor 7); and encouraging the staff to take ownership of the EBP (Factor 8). The scale is answered on a five-point Likert scale (1 = *scarcely applicable* to 5 = *highly applicable*). The mean scores of each ward were calculated for all eight factors of the GOLA Inventory to identify the specific activities that promote nurses' internalization of EBPs. Cronbach's alpha scores for the factors ranged from 0.82 to 0.95. To enable respondents to assess the extent of their ward's organizational learning activities for the selected EBP, the instructions indicated, “You are asked these questions to elaborate on the activities or efforts that have been implemented in your department/ward to date since the introduction of (name of the EBP). The EBP mentioned in the questions refers to the (name of the EBP) implemented in the unit.”

#### 2.2.3. Control Variables

The individual characteristics associated with the internalization of EBPs were as follows: (1) nurses' ability to solve patients' problems, which was measured using the Problem-Solving Client Support Behavior Self-Evaluation Scale for Nurses [28]; (2) Japanese version of EBP beliefs scale [29], which was based on the EBP Belief Scale [30], for assessing individuals' beliefs about the value of, and their ability to perform the EBP; and (3) other factors, such as age, years of working as a nurse, years of working in the current hospital, years of working in the current department/ward, education level, experience in intrahospital transfer, and experience in nursing research.

Further, the Japanese version of the Practice Environment Scale of the Nursing Work Index (PES-NWI) [31] was used to measure practice environments. As the duration of exposure to organizational learning activities from the introduction of an EBP into the department/ward to the time of the survey may influence an individual's internalization of EBPs, the researchers asked those in charge in the participating hospitals when the target EBP was introduced into each department/ward. Finally, the type of EBP was included—namely, whether the EBP was included in the current medical fee system in Japan.

### 2.3. Data Analyses

#### 2.3.1. Preanalyses

We examined the organizational learning activities at the department/ward level. One method for evaluating a group is to calculate the mean score of the group based on the scores of members' evaluations of the group to which they belong [32]. To confirm the validity of using group-level variables, ICC (1), ICC (2) [33], and the within-group agreement index (rWG) [32, 34] were calculated. Significantly, the primary analysis showed that ICC (1) was 0.18, ICC (2) was 0.73, and rWG was 0.59 (range: 0.18–1.00). Therefore, we used the mean score of the organizational learning activities at the department/ward level as a proxy of the organizational learning activities.

Intraclass correlation coefficients were calculated for the null model with the internalization of EBPs as the objective variable. The intercept variance of the internalization of EBPs was 31% (95% CI = 0.15, 0.63) with ICC (1) being 0.14. As departments or wards influenced 14% of nurses' internalization of EBPs, we selected hierarchical linear modeling (HLM) and performed it with fixed effects using the maximum likelihood method [35].

To select control variables, we assessed correlation coefficients between the characteristics of individuals and wards as they relate to the internalization of EBPs. We used the Mann–Whitney *U*-test for the categorical data.

#### 2.3.2. Main Analyses

As we hypothesized that differences in organizational learning activities by ward affect nurses' EBP internalization, we adopted the centering within the cluster-mean (CWC-M) model. The objective variable was the internalization of EBPs, while the explanatory variable was the GOLA Inventory score (CWC) for the individual level and GOLA Inventory score (ward mean) for the ward/department level; the control variables were significantly associated with the internalization of EBPs in the bivariate analyses. This study aimed to identify the contextual effect [36] which is the direct ward-level effect without an individual's perception of the organizational learning activities at the ward/department level. For that reason, on nurses' internalization of EBPs, we subtracted the coefficient of the individual-level explanatory variable calculated for each model from the coefficient of the ward-level explanatory variable. The significance level was *p* < 0.05. IBM SPSS Statistics Version 27 was used for the statistical analysis.

### 2.4. Ethical Considerations

We explained the purpose, method, risks, and benefits of the study to the participants. We informed them that their participation was voluntary and that their decision not to participate would not disadvantage them. Before administering the online questionnaire, we provided participants with the URL and QR codes to log into the website, which stated that they could not be identified by their login ID. All participants consented to their inclusion in the survey before the procedure. The frontline nursing manager of the respective departments distributed the research materials to each nurse. To reduce coercion, we asked nursing managers to explain to all nurses that their participation was not a work task and that their decision or refusal to participate would not affect their work evaluation. Nurses who participated in the survey received an Amazon gift card (e-mail type) of 300 yen. This study was approved by the Research Ethics Committee of the Graduate School of Medicine, the University of Tokyo (2021131NI).

## 3. Results

### 3.1. Participants and Measurements

We used the means of the wards' organizational learning activities to evaluate the group-level variables [32]. Wards with two or fewer valid responses were excluded from the analysis. Ishii, Takemura, and Ichikawa have provided details on the sampling, hospital characteristics (Table 3 in [23]), and the characteristics of wards and participants (Table 2 in [22]). The functions of the participating hospitals were regional medical-care support (75.0%) and advanced treatment (16.7%). The average number of beds in these hospitals was 615.7 (standard deviation [SD] = 154.2). Types of clinical departments of wards were mixed (31.1%), internal medicine (29.2%), and surgery (22.9%). The average number of valid responses in each ward was 7.5, and the average duration from the introduction of the EBP to the survey was 13.8 months (SD = 2.8).

### 3.2. Main Analysis

The following characteristics showed significant correlations with the internalization of EBPs (Table 1): years of working as a nurse (*ρ* = 0.19), years of working in the current hospital (*ρ* = 0.22), years of working in the current ward/department (*ρ* = 0.26), problem-solving (*ρ* = 0.41), EBP belief (*ρ* = 0.39), eight factors in the GOLA Inventory (individual level) (*ρ* = 0.39–0.49), eight factors in the GOLA Inventory (ward level) (*ρ* = 0.25–0.30), and practice environments (PES-NWI) (Factor 1, Factor 3: *ρ* = 0.14, 0.20). A Mann–Whitney *U*-test conducted to measure nurses' internalization of EBPs showed significant differences by education level (*U* = 14020,00, *p* = 0.029), experience of intrahospital transfer (*U* = 12,488.00, *p* < 0.001), and the type of EBP (*U* = 13,827.50, *p* = 0.016). Among the control variables, years of working in the current hospital, education level, problem-solving, and EBP belief were used as individual characteristics, whereas the type and duration of EBP and practice environments (five factors) were used as group characteristics. Since a correlation was observed between the participants' years of working as nurses and years of working in the current hospital (*ρ* = 0.90), the latter was used.

The HLM results showed that all eight factors in the GOLA Inventory (ward level) were positively and significantly associated with nurses' internalization of EBPs (*β* = 0.160–0.354, 95% CI: [0.041, 0.279]∼[0.212, 0.496]) (Table 2). The contextual effects of the eight factors in the GOLA Inventory were −0.003 for Factor 1, 0.009 for Factor 2, 0.073 for Factor 3, 0.140 for Factor 4, 0.125 for Factor 5, 0.067 for Factor 6, 0.120 for Factor 7, and 0.138 for Factor 8.

## 4. Discussion

This study verified that all eight factors in the GOLA Inventory were associated with nurses' internalization of the target EBPs. In a study that treated GOLA as an entire ward activity, GOLA was not significantly associated with nurses' internalization of EBPs [22]. We found that the contextual effects of the organizational learning activities at the ward/department level on the nurses' internalization of EBPs were different among the eight factors. In other words, increasing nurses' internalizations of EBP may require more strategic activities than mere learning activities conducted in entire wards, which is the recommendation based on the findings of this secondary analysis.

### 4.1. Organizational Learning Activities at the Ward/Department Level Are Weakly Related to Nurses' Internalization of EBPs

Although Factor 1—organizing a team to lead the EBP in the unit—was positively and significantly associated with nurses' internalization of EBPs, its contextual effect on their internalization was extremely small. Previous studies have shown that forming a powerful guiding coalition is essential for the successful transformation of organizations [37]. Furthermore, creating clinical teams for collaborative learning is included in the implementation strategy of EBPs [38]. To improve attitudes, knowledge, and practices related to EBPs, however, not only forming groups but also annual audits of projects and having internal and external facilitators are important [39]. We found that forming a team within a department had little effect on nurses' internalization of EBPs. When starting a new EBP, many hospitals first organize EBP teams. However, this study showed that despite efforts to create EBP teams within wards, nurses' internalization was not particularly improved; therefore, congruent with previous research, the mere organization of an EBP team is not sufficient.

Factor 2—evaluating the implemented EBP from multiple angles—was positively and significantly associated with nurses' internalization of EBPs. However, its contextual effect was extremely small. Previous studies have suggested that group discussions and others' evaluations can facilitate educators' internalization of knowledge [40]. Similarly, items in Factor 2 included “holding conferences regularly to evaluate whether the implemented EBP is appropriate” and “regularly discussing the implemented EBP with other professionals and multidisciplinary teams.” Therefore, we assumed that by conducting conferences and meetings related to EBPs at the ward and providing opportunities and venues for multidisciplinary discussions, nurses would enhance their understanding of the significance of EBPs. However, simply holding discussions and conferences on EBPs within departments may not foster nurses' internalization of EBPs because, even at a multidisciplinary conference, individual nurses may not participate in the discussion cognitively.

Factor 3—ensuring that staff can acquire common knowledge of the EBP—was positively and significantly associated with nurses' internalization of EBPs. However, the contextual effect of Factor 3 was extremely small. Previous studies have shown that senders' ability in the communication process is important for the internalization of knowledge [41]. For each staff member to acquire common organizational knowledge, managers must repeatedly explain the concept to each nurse in multiple ways to ensure their understanding, which may encourage the individual to internalize the EBP.

The contextual effect of Factor 6—sharing the significance of EBP implementation with the unit and staff—was extremely small. Qualitative research on healthcare knowledge management suggests that patients' and providers' efforts to make a common sense of a disease may promote the internalization of the disease [42]. However, this study suggests that nurses' internalization of EBPs may not be fostered when they are simply told that the practice is important or significant.

These activities may not include the cognitive participation of nurses who perform EBPs. This may explain why these activities did not demonstrate a strong relationship with the internalization of EBPs, which is a cognitive change in understanding the value and significance of EBPs.

### 4.2. Organizational Learning Activities at the Ward/Department Level Are Relatively Highly Related to Nurses' Internalization of EBPs

The contextual effect of Factor 4—ensuring that staff can understand why the EBP is being implemented—was higher than that of the previous factors. Studies have reported the potential influence of collective sense-making work, which involves efforts to differentiate newly introduced practices favorably from preexisting practices (differentiation) when new practices are implemented [43]. Factor 4 included items explaining the benefits and drawbacks of implementing EBPs and encompassing ward-wide efforts to understand the significance of engaging in EBPs. Previous studies have demonstrated the direct impact of collective sense-making on EBP implementation. We found that activities designed to foster an understanding of the reasons for implementing EBPs also influenced nurses' internalization of EBPs.

The contextual effect of Factor 5—ensuring that staff can implement the EBP in a unified manner—was also higher than that of the other factors. Prior research has shown that when managers verify that employees follow procedures, create rules for them to conform to new standards of practice, and modify existing procedures, the employees effectively internalize the practices [43]. Factor 5 contains similar items, such as establishing a uniform method of implementation (e.g., formulating EBP rules and creating procedures). Communicating during this process and explaining the procedures being created may lead to an understanding of the significance of the EBP, which is the basis of behavioral change.

The contextual effect of Factor 7—encouraging staff to better implement the EBP within the unit—was higher than that of the other factors. This factor included staff's consulting with managers regarding EBPs, proposing involvement in EBPs according to each staff member's career progression, and ensuring appropriate staffing of nurses for the implementation of EBPs. Previous studies have not identified these factors as internalization factors. This study revealed that engaging in these activities at the ward level was associated with nurses' internalization of EBPs. Future research is required to examine why these factors are related to the internalization of EBPs.

The contextual effect of Factor 8—encouraging staff to take ownership of the EBP—was higher than that of the other factors. Prior research has indicated that assigning specific roles to staff members and holding each person accountable for EBPs [44] are effective in establishing EBPs. In addition, the nurses' professional autonomy is related to their patient safety activities [45]. In other words, previous studies have shown that assigning responsibility and fostering autonomy influence EBP and nursing practice. Factor 8 included similar activities meant to give individuals in each ward the discretion to implement EBPs and make all members perceive EBPs as their own responsibilities. However, this study suggested that these activities might promote the understanding of the significance and value of EBP among the nurses in that department. Therefore, this study found that activities in the entire department contribute to nurses' awareness of the internalization of EBPs.

We revealed several ways for nurses to foster the internalization of EBPs when they are introduced by hospitals. First, it is important to undertake efforts to encourage staff to understand why an EBP is being implemented. Next, it is essential to create rules and procedures in the ward so that the EBP can be implemented in a unified manner. Finally, staff must be encouraged to implement the EBPs well within the ward and take ownership of the EBP.

### 4.3. Limitations and Strengths

This study has some limitations. First, this study used the same dataset as the psychometric validation study [23], and there was some overlap in sampling for some participants. Concerns about the GOLA Inventory's validity and the results' reliability exist. To prevent shortcomings of the primary research measurements [24], individual characteristics (e.g., EBP belief) and organizational characteristics (e.g., PES-NWI) were used as control variables. By performing a secondary analysis of the primary research data, we can analyze data from a group appropriate for the purpose without deviations from the core measurements in the secondary analysis. We considered gathering more participants, but the number of participants was determined after invitations for participation were sent to over 200 hospitals nationwide, so we thought additional requirements would be difficult. Because this is a secondary analysis, future research should verify the reliability of the results by targeting different hospitals and EBPs.

Second, some nurses might already have a positive attitude toward EBPs. Since 2017, the Japanese Ministry of Education, Culture, Sports, Science and Technology has advocated the acquisition of knowledge on “evidence-based problem-solving skills” and “scientific inquiry” in bachelor's degree programs for nursing [46]. As the nurses who participated in this study were young and almost half (45.8%) had graduated from university, many may have had high EBP literacy from the start of their nursing careers.

Third, this cross-sectional study assessed the group-level organizational learning activities in wards from the introduction of the EBPs in those wards to the time that the survey was conducted. Since the average duration of this period was 13 months, we may have assigned some respondents to wards after the introduction of the EBP. As the level of those activities was likely to have been high for the first several months after the introduction of the EBPs, some nurses who participated in this study may not have experienced these activities at all or may have evaluated the period when the level of activities was low.

Fourth, the use of self-reported data for all the variables may have increased the correlation between the measured variables.

Finally, most of the wards that participated in this study were surgical or internal medicine wards. Psychiatry or maternity wards were excluded. Furthermore, the types of the targeted EBPs were also limited. Therefore, the findings of this study should be interpreted with caution, as their generalizability is limited.

These findings will help nurse managers and nursing educators understand how they should develop support systems and educational programs based on organizational learning theory that can promote nurses' internalization of EBPs in hospitals. In addition, hospitals or wards implementing EBPs can adjust their organizational learning activities based on our results and select those that contribute the most to nurses' internalization of EBPs. Although internalization is crucial to the prevention of superficial practices, specific activities to enhance the internalization of EBPs in nursing have not yet been clarified. This study contributes to nursing science and implementation science by clarifying that group-level organizational learning activities can enhance the internalization of EBPs.

### 4.4. Recommendations for Further Research

Future research should improve the study design in various ways, such as asking questions that limit the response period to times when the degree of activities is high, conducting longitudinal studies to clarify the causal relationships, and measuring changes in internalization through intervention designs. It is also necessary to examine the effects of the internalization of EBPs toward actual practice compared to EBP attitudes and EBP beliefs. A better method for measuring group-level organizational learning activities rather than using self-reported scores (e.g., using third-party evaluation scores) should also be considered.

## 5. Conclusions

This study examined the relationship between the eight factors in the GOLA Inventory, which is used to measure the organizational learning activities within a hospital ward in the feedback process of the organizational learning model, and the implementation of and nurses' internalization of EBPs. Furthermore, it studied the contextual effects (i.e., effective concrete aspects) of each group learning activity on the internalization of EBPs. The findings revealed the ward-level activities that are necessary for nurses to internalize the significance of EBPs, including ensuring that the staff can understand why a specific EBP is being implemented, they can implement the EBP in a unified way, they are encouraged to better implement the EBP within the unit, and they are encouraged to take ownership of the EBP. Wards in which EBPs are implemented can set the backdrop for nurses' internalization of EBPs and could prevent their superficial practice by engaging them in group-level organizational learning activities.

## Figures and Tables

**Table 1:** Scores of variables and Spearman's rank correlation coefficients (*n* = 360, 48 wards).

		**Mean ± SD**	**1**	**2**	**3**	**4**	**5**	**6**	**7**	**8**	**9**	**10**	**11**	**12**	**13**	**14**	**15**

*Individual level*																	
1	Age	32.8 ± 9.8	—														
2	Years of working as a nurse	10.3 ± 9.3	0.96⁣^∗∗^	—													
3	Years of working in the current hospital	8.7 ± 8.5	0.87⁣^∗∗^	0.90⁣^∗∗^	—												
4	Years of working in the current ward/department	3.7 ± 3.9	0.32⁣^∗∗^	0.33⁣^∗∗^	0.41⁣^∗∗^	—											
5	Problem-solving	3.9 ± 0.5	0.23⁣^∗∗^	0.26⁣^∗∗^	0.24⁣^∗∗^	0.15⁣^∗∗^	—										
6	EBP belief	3.2 ± 0.4	0.09	0.12⁣^∗^	0.14⁣^∗∗^	0.13⁣^∗^	0.29⁣^∗∗^	—									
7	GOLA_1	2.9 ± 1.2	0.09	0.11⁣^∗^	0.11⁣^∗^	−0.01	0.20⁣^∗∗^	0.28⁣^∗∗^	—								
8	GOLA_2	2.9 ± 1.0	0.06	0.09	0.11⁣^∗^	−0.01	0.25⁣^∗∗^	0.32⁣^∗∗^	0.71⁣^∗∗^	—							
9	GOLA_3	3.0 ± 0.9	0.11⁣^∗^	0.14⁣^∗∗^	0.17⁣^∗∗^	0.01	0.25⁣^∗∗^	0.32⁣^∗∗^	0.68⁣^∗∗^	0.80⁣^∗∗^	—						
10	GOLA_4	2.8 ± 1.0	0.01	0.03	0.06	−0.03	0.21⁣^∗∗^	0.32⁣^∗∗^	0.65⁣^∗∗^	0.78⁣^∗∗^	0.81⁣^∗∗^	—					
11	GOLA_5	2.9 ± 1.0	0.03	0.05	0.09	−0.02	0.24⁣^∗∗^	0.32⁣^∗∗^	0.64⁣^∗∗^	0.74⁣^∗∗^	0.83⁣^∗∗^	0.86⁣^∗∗^	—				
12	GOLA_6	2.8 ± 1.0	0.01	0.03	0.07	−0.02	0.23⁣^∗∗^	0.30⁣^∗∗^	0.61⁣^∗∗^	0.72⁣^∗∗^	0.75⁣^∗∗^	0.82⁣^∗∗^	0.86⁣^∗∗^	—			
13	GOLA_7	2.7 ± 1.0	−0.01	0.01	0.05	−0.05	0.24⁣^∗∗^	0.33⁣^∗∗^	0.57⁣^∗∗^	0.75⁣^∗∗^	0.73⁣^∗∗^	0.77⁣^∗∗^	0.81⁣^∗∗^	0.87⁣^∗∗^	—		
14	GOLA_8	2.7 ± 1.0	−0.02	−0.01	0.03	−0.03	0.20⁣^∗∗^	0.29⁣^∗∗^	0.50⁣^∗∗^	0.66⁣^∗∗^	0.66⁣^∗∗^	0.72⁣^∗∗^	0.75⁣^∗∗^	0.77⁣^∗∗^	0.86⁣^∗∗^	—	
15	Internalization of EBPs	3.4 ± 0.8	0.19⁣^∗∗^	0.22⁣^∗∗^	0.26⁣^∗∗^	0.04	0.41⁣^∗∗^	0.39⁣^∗∗^	0.39⁣^∗∗^	0.46⁣^∗∗^	0.49⁣^∗∗^	0.45⁣^∗∗^	0.47⁣^∗∗^	0.42⁣^∗∗^	0.44⁣^∗∗^	0.39⁣^∗∗^	—

*Ward level*																	
16	Duration of the EBP (months)	13.8 ± 2.8	0.13⁣^∗^	0.12⁣^∗∗^	0.12⁣^∗^	0.12⁣^∗^	0.12⁣^∗^	−0.05	0.10	0.08	0.00	0.06	−0.01	0.06	0.04	0.03	0.14⁣^∗∗^
17	Number of nurses in the ward	30.2 ± 7.4	−0.03	−0.04	−0.02	−0.00	0.01	0.01	−0.13⁣^∗^	−0.10	−0.10	−0.06	−0.06	−0.05	−0.02	0.01	0.01
18	PES-NWI_1	2.3 ± 0.2	0.15⁣^∗∗^	0.14⁣^∗∗^	0.10	−0.01	0.08	−0.06	0.11⁣^∗^	0.06	0.03	0.01	0.04	0.01	0.05	0.02	0.14⁣^∗∗^
19	PES-NWI_2	2.1 ± 0.2	0.11⁣^∗^	0.09	0.05	−0.01	−0.01	−0.04	0.10	0.04	0.01	−0.01	0.01	0.02	0.02	0.02	0.02
20	PES-NWI_3	2.2 ± 0.4	0.15⁣^∗∗^	0.15⁣^∗∗^	0.100	−0.01	0.15⁣^∗∗^	−0.02	0.07	0.04	0.01	0.02	0.02	0.03	0.08	0.03	0.20⁣^∗∗^
21	PES-NWI_4	2.6 ± 0.3	0.09	0.06	0.01	−0.10	−0.04	−0.06	0.06	−0.08	−0.06	−0.06	−0.01	−0.05	−0.03	−0.05	−0.03
22	PES-NWI_5	2.2 ± 0.3	0.16⁣^∗∗^	0.15⁣^∗∗^	0.12⁣^∗^	−0.05	0.01	−0.01	0.15⁣^∗∗^	0.15⁣^∗∗^	0.11⁣^∗^	0.07	0.12⁣^∗^	0.07	0.13⁣^∗^	0.11⁣^∗^	0.07
23	GOLA_1_ward mean	2.9 ± 0.7	0.10	0.11⁣^∗^	0.12⁣^∗^	−0.04	0.06	0.09	0.60⁣^∗∗^	0.47⁣^∗∗^	0.42⁣^∗∗^	0.42⁣^∗∗^	0.45⁣^∗∗^	0.44⁣^∗∗^	0.40⁣^∗∗^	0.37⁣^∗∗^	0.26⁣^∗∗^
24	GOLA_2_ward mean	2.9 ± 0.6	0.08	0.10	0.11⁣^∗^	−0.01	0.06	0.10⁣^∗^	0.48⁣^∗∗^	0.58⁣^∗∗^	0.46⁣^∗∗^	0.44⁣^∗∗^	0.43⁣^∗∗^	0.46⁣^∗∗^	0.43⁣^∗∗^	0.41⁣^∗∗^	0.25⁣^∗∗^
25	GOLA_3_ward mean	3.0 ± 0.5	0.13⁣^∗^	0.15⁣^∗∗^	0.17⁣^∗∗^	0.02	0.06	0.13⁣^∗^	0.46⁣^∗∗^	0.50⁣^∗∗^	0.53⁣^∗∗^	0.42⁣^∗∗^	0.46⁣^∗∗^	0.45⁣^∗∗^	0.42⁣^∗∗^	0.41⁣^∗∗^	0.30⁣^∗∗^
26	GOLA_4_ward mean	2.8 ± 0.6	0.04	0.07	0.10	0.01	0.02	0.10	0.49⁣^∗∗^	0.51⁣^∗∗^	0.44⁣^∗∗^	0.50⁣^∗∗^	0.48⁣^∗∗^	0.49⁣^∗∗^	0.46⁣^∗∗^	0.45⁣^∗∗^	0.30⁣^∗∗^
27	GOLA_5_ward mean	2.9 ± 0.5	0.08	0.11⁣^∗^	0.13⁣^∗^	−0.04	0.04	0.12⁣^∗^	0.52⁣^∗∗^	0.49⁣^∗∗^	0.47⁣^∗∗^	0.47⁣^∗∗^	0.53⁣^∗∗^	0.47⁣^∗∗^	0.45⁣^∗∗^	0.43⁣^∗∗^	0.30⁣^∗∗^
28	GOLA_6_ward mean	2.8 ± 0.5	0.06	0.09	0.11⁣^∗^	0.01	0.04	0.10	0.51⁣^∗∗^	0.53⁣^∗∗^	0.46⁣^∗∗^	0.48⁣^∗∗^	0.48⁣^∗∗^	0.51⁣^∗∗^	0.47⁣^∗∗^	0.44⁣^∗∗^	0.28⁣^∗∗^
29	GOLA_7_ward mean	2.7 ± 0.6	0.04	0.07	0.091	−0.01	0.06	0.12⁣^∗^	0.46⁣^∗∗^	0.51⁣^∗∗^	0.44⁣^∗∗^	0.46⁣^∗∗^	0.46⁣^∗∗^	0.48⁣^∗∗^	0.50⁣^∗∗^	0.44⁣^∗∗^	0.30⁣^∗∗^
30	GOLA_8_ward mean	2.7 ± 0.5	0.05	0.07	0.105	−0.01	0.03	0.10	0.45⁣^∗∗^	0.50⁣^∗∗^	0.44⁣^∗∗^	0.46⁣^∗∗^	0.47⁣^∗∗^	0.46⁣^∗∗^	0.45⁣^∗∗^	0.48⁣^∗∗^	0.27⁣^∗∗^

		**16**	**17**	**18**	**19**	**20**	**21**	**22**	**23**	**24**	**25**	**26**	**27**	**28**	**29**	**30**	

*Ward level*																	
16	Duration of the EBP	—															
17	Number of nurses in the ward	−0.10	—														
18	PES-NWI_1	0.23⁣^∗∗^	−0.11⁣^∗^	—													
19	PES-NWI_2	0.15⁣^∗∗^	−0.02	0.85⁣^∗∗^	—												
20	PES-NWI_3	0.35⁣^∗∗^	0.11⁣^∗^	0.74⁣^∗∗^	0.61⁣^∗∗^	—											
21	PES-NWI_4	0.05	−0.01	0.66⁣^∗∗^	0.72⁣^∗∗^	0.44⁣^∗∗^	—										
22	PES-NWI_5	0.02	−0.12⁣^∗^	0.67⁣^∗∗^	0.73⁣^∗∗^	0.45⁣^∗∗^	0.64⁣^∗∗^	—									
23	GOLA_1_ward mean	0.20⁣^∗∗^	−0.21⁣^∗^	0.20⁣^∗∗^	0.12⁣^∗∗^	0.12⁣^∗^	0.11⁣^∗^	0.23⁣^∗∗^	—								
24	GOLA_2_ward mean	0.08	−0.11⁣^∗^	0.02	0.02	0.04	−0.20⁣^∗^	0.20⁣^∗∗^	0.77⁣^∗∗^								
25	GOLA_3_ward mean	−0.06	−0.17⁣^∗^	0.01	0.00	−0.02	−0.10	0.18⁣^∗∗^	0.74⁣^∗∗^	0.81⁣^∗∗^	—						
26	GOLA_4_ward mean	0.13⁣^∗^	−0.03	−0.01	−0.04	0.05	−0.13⁣^∗^	0.08	0.80	0.84⁣^∗∗^	0.78⁣^∗∗^	—					
27	GOLA_5_ward mean	−0.03	−0.09	0.03	−0.02	0.02	−0.05	0.17⁣^∗∗^	0.85⁣^∗∗^	0.80⁣^∗∗^	0.85⁣^∗∗^	0.91⁣^∗∗^	—				
28	GOLA_6_ward mean	0.09	−0.05	0.01	−0.01	0.06	−0.15⁣^∗^	0.10	0.83⁣^∗∗^	0.89⁣^∗∗^	0.84⁣^∗∗^	0.95⁣^∗∗^	0.90⁣^∗∗^	—			
29	GOLA_7_ward mean	0.06	−0.01	0.09	0.06	0.16⁣^∗∗^	−0.07	0.19⁣^∗∗^	0.75⁣^∗∗^	0.83⁣^∗∗^	0.79⁣^∗∗^	0.89⁣^∗∗^	0.86⁣^∗∗^	0.91⁣^∗∗^	—		
30	GOLA_8_ward mean	0.07	0.09	0.01	0.01	0.04	−0.12⁣^∗^	0.13⁣^∗^	0.74⁣^∗∗^	0.82⁣^∗∗^	0.79⁣^∗∗^	0.92⁣^∗^	0.87⁣^∗∗^	0.89⁣^∗∗^	0.87⁣^∗∗^	—	

*Note:* Spearman's rank correlation, ⁣^∗^*p* < 0.05, ⁣^∗∗^*p* < 0.01, ⁣^∗∗∗^*p* < 0.001, no.1: age; no. 2: years of working as a nurse; no. 3: years of working in the current hospital; no. 4: years of working in the current ward/department; no. 5: problem-solving behavior: Problem-Solving Client-Support Behavior Self-Evaluation Scale for Nurses (range: 1–5); no. 6: EBP belief: the Japanese Version of Evidence-Based Practice Belief Scale (range: 1–5); no. 7∼no. 14: GOLA Inventory factor_1–8 (individual level)(range: 1–5); no. 15: internalization of EBP, PES-NWI: the Practice Environment Scale of the Nursing Work Index (range: 1–5).

Abbreviations: GOLA = group organizational learning activity inventory, SD = standard deviation.

**Table 2:** Individual- and ward-level analyses of nurses' internalization of EBPs using two-level HLM (*n* = 360, 48 wards).

	Internalization of EBPs				
	*β*	SD	*p*	95% CI	
				Low	Upper
Intercept	0.200	0.577	0.730	−0.950	1.350

*Individual level*					
GOLA Inventory factor (CWC)					
1. Organizing a team to lead the EBP in the unit	0.163	0.034	< 0.001	0.096	0.231
2. Evaluating the implemented EBP from multiple angles	0.267	0.043	< 0.001	0.183	0.350
3. Ensuring that staff can acquire common knowledge of the EBP	0.252	0.042	< 0.001	0.169	0.335
4. Ensuring that staff can understand why the EBP is being implemented	0.187	0.038	< 0.001	0.112	0.261
5. Ensuring that staff can implement the EBP in a unified manner	0.229	0.041	< 0.001	0.148	0.310
6. Sharing the significance of EBP implementation with the unit and staff	0.193	0.040	< 0.001	0.113	0.272
7. Encouraging staff to better implement the EBP within the unit	0.173	0.038	< 0.001	0.098	0.247
8. Encouraging staff to take ownership of the EBP	0.155	0.038	< 0.001	0.081	0.230

*Ward level*					
GOLA Inventory factor (ward-mean)					
1. Organizing a team to lead the EBP in the unit	0.160	0.059	0.010	0.041	0.279
2. Evaluating the implemented EBP from multiple angles	0.276	0.073	< 0.001	0.128	0.424
3. Ensuring that staff can acquire common knowledge of the EBP	0.325	0.071	< 0.001	0.181	0.469
4. Ensuring that staff can understand why the EBP is being implemented	0.327	0.069	< 0.001	0.188	0.466
5. Ensuring that staff can implement the EBP in a unified manner	0.354	0.072	< 0.001	0.212	0.496
6. Sharing the significance of EBP implementation with the unit and staff	0.260	0.073	< 0.001	0.112	0.407
7. Encouraging staff to better implement the EBP within the unit	0.293	0.070	< 0.001	0.151	0.434
8. Encouraging staff to take ownership of the EBP	0.293	0.070	< 0.001	0.151	0.434

*Contextual effects of the eight factors in the GOLA Inventory*					
1. Organizing a team to lead the EBP in the unit	−0.003				
2. Evaluating the implemented EBP from multiple angles	0.009				
3. Ensuring that staff can acquire common knowledge of the EBP	0.073				
4. Ensuring that staff can understand why the EBP is being implemented	0.140				
5. Ensuring that staff can implement the EBP in a unified manner	0.125				
6. Sharing the significance of EBP implementation with the unit and staff	0.067				
7. Encouraging staff to better implement the EBP within the unit	0.120				
8. Encouraging staff to take ownership of the EBP	0.138				

*Note:* The control variables were years of working in the current hospital, education level, problem-solving, EBP belief, type of EBP, duration of EBP, and practice environments (five factors). The eight factors of the GOLA Inventory were added into separate models one factor at a time. The residual, variance of intercept, and Akaike information criterion of each model were Model 1 = 0.346, 0.014, 705.23; Model 2 = 0.329, 0.010, 684.64; Model 3 = 0.332, 0.005, 683.88; Model 4 = 0.343, 0.001, 692.36; Model 5 = 0.338, 0.000, 685.99; Model 6 = 0.345, 0.010, 700.39; Model 7 = 0.347, 0.004, 698.97; and Model 8 = 0.353, 0.001, 702.81.

Abbreviations: HLM, hierarchical linear model; SE, standard error.

## Data Availability

The datasets analyzed during the current study are not publicly available due to ethical restrictions.
